# A Rare Confluence: Brain Abscess in an Adult With Tetralogy of Fallot

**DOI:** 10.7759/cureus.63860

**Published:** 2024-07-04

**Authors:** Palak Gupta, Harpratap Singh, Naveya Vashisht, Gurneet Singh Dhingra, Vanshdeep Sharma

**Affiliations:** 1 Medicine, Maharishi Markandeshwar Institute of Medical Sciences and Research, Ambala, IND; 2 Medicine, Subharti Medical College, Meerut, IND

**Keywords:** neurological confluence, brain abscess, unrepaired tetralogy of fallot, cyanotic congenital heart disease, congenital heart disease

## Abstract

Tetralogy of Fallot (TOF) is a common congenital heart disease (CHD) characterized by four distinct cardiac abnormalities. Brain abscess, though rare, is a life-threatening complication in patients with cyanotic congenital heart disease (CCHD), including TOF. This case report describes a 24-year-old female with unrepaired TOF who presented with symptoms of a brain abscess, including altered sensorium, fever, projectile vomiting, and headache. Diagnostic imaging with non-contrast-enhanced computed tomography (NCCT) revealed a well-defined hypodense lesion with a midline shift, prompting urgent drainage of the abscess. Subsequent cultures of the pus material identified *Streptococcus intermedius* as the causative agent, and the patient was maintained on antibiotics. This case highlights the importance of early diagnosis and surgical repair of TOF to prevent severe complications such as brain abscess, thereby reducing morbidity and mortality.

## Introduction

Congenital heart disease (CHD), the most common type of congenital disorder, represents structural abnormalities of the heart or intrathoracic great vessels that occur during fetal development [1]. It is seen in approximately 1% to 2% of live births and it is the leading cause of death in children born with congenital abnormalities [1,2]. In India, with a birth prevalence of nine in 1000, more than 200000 children are born with CHD each year [3]. CHD can further be divided into cyanotic congenital heart disease (CCHD) and non-CCHD. Tetralogy of Fallot (TOF) is the most common CHD, with a prevalence of one out of 3000 births, and an incidence of five to seven out of 10000 live births thereby representing 5% to 7% of all CHD [4]. TOF having nearly equal sex distribution is characterized by four defects: obstruction of the right ventricular outflow tract, enlargement of the right ventricle, ventricular septal defect, and aortic overriding [5]. Due to advancements in medicine and surgery, the prevalence of CHD has increased in older adults and children. Approximately one million adults in the US have CHD, with 15% of those patients being treated for TOF [6]. On the other hand, in patients with unrepaired TOF, survival decreases as the patient ages, estimated survival rate to be 66% at one year of age, 40% at three years, 11% at 20 years, 6% at 30 years, and 3% at 40 years [4].

Brain abscess, a rare but potentially life-threatening infection of the brain parenchyma, can be associated with CCHD. TOF is present in 5-18.7% of CCHD patients with cerebral abscesses [7]. Depending on the size and location of the abscess, surrounding edema, and virulence of the infectious agent, the clinical presentation of individuals with brain abscesses varies. Common symptoms include headache, projectile vomiting, seizures, focal neurological deficits, altered sensorium, and fever [8].

In a developing country like India, early detection and repair of TOF is crucial to prevent serious life-threatening complications such as brain abscesses. This case report highlights a 24-year-old female with unrepaired TOF, who presented with a brain abscess, emphasizing the necessity of early diagnosis and management.

## Case presentation

A 24-year-old female presented to the emergency department with complaints of altered sensorium for three days, along with two episodes of projectile vomiting, high-grade fever, and headache for seven days. She had a history of recurrent episodes of shortness of breath since childhood and was diagnosed with TOF through echocardiography in infancy but did not undergo any surgical correction due to financial problems. 

On examination, she was disoriented to time, place, and person with a Glasgow Coma Scale (GCS) score of 09/15. Physical examination revealed clubbing and cyanosis, a temperature of 101 °F, a pulse rate of 120 beats/minute, blood pressure of 130/80 mmHg, a respiratory rate of 22 breaths/minute, and SpO_2_ varying between 80% and 85% in room air. A harsh, systolic ejection murmur, grade 3/6 was audible at the left second intercostal space during cardiac examination. The remainder of the systemic examination was within normal limits. 

**Figure 1:**
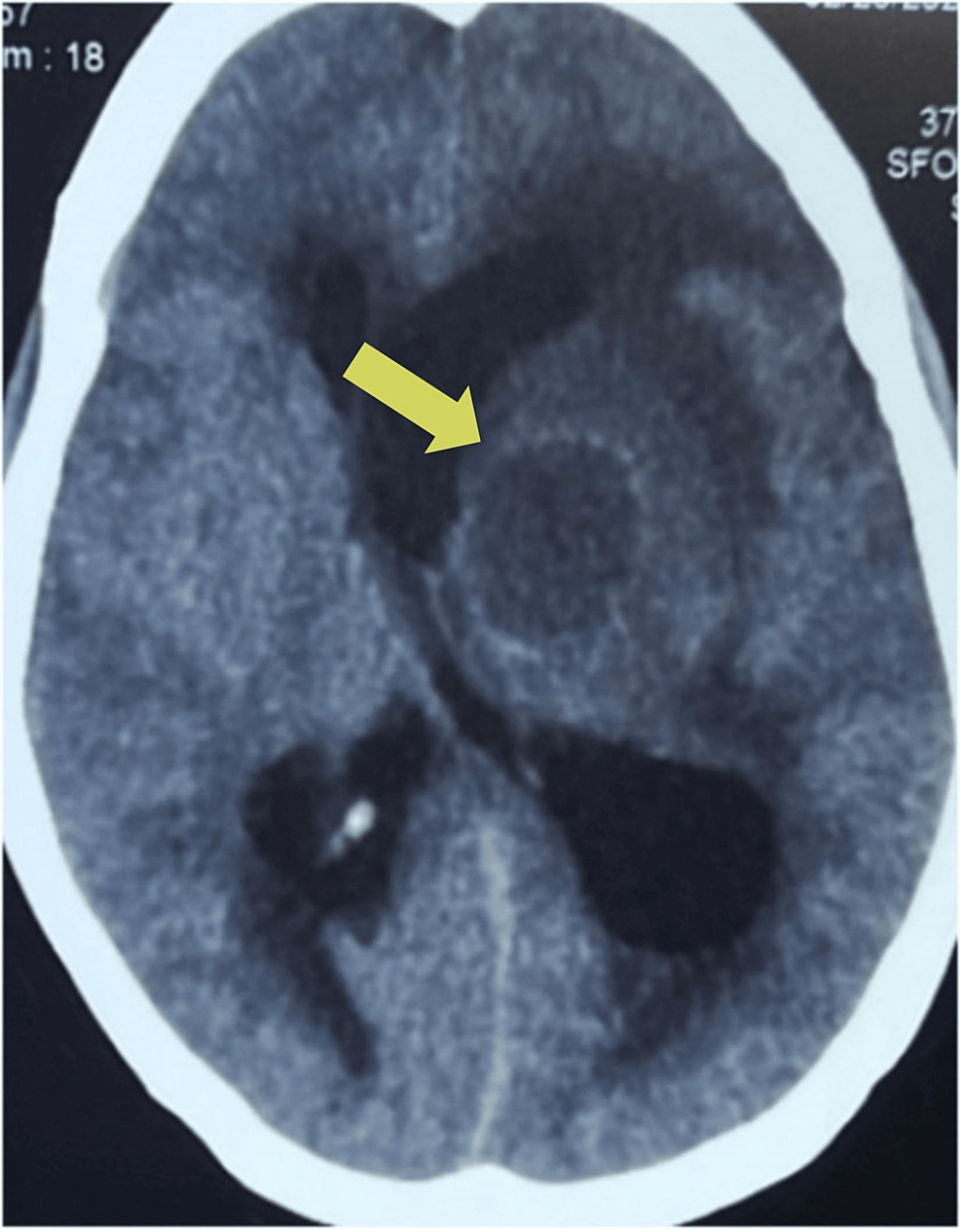
NCCT brain showing a 23.8×23.5×27.6 mm well-defined hypodense lesion in the left perisylvian region and thalamus with surrounding cerebral edema causing midline shift toward the right with contralateral compression of the right lateral ventricle. NCCT, non-contrast-enhanced computed tomography

**Figure 2:**
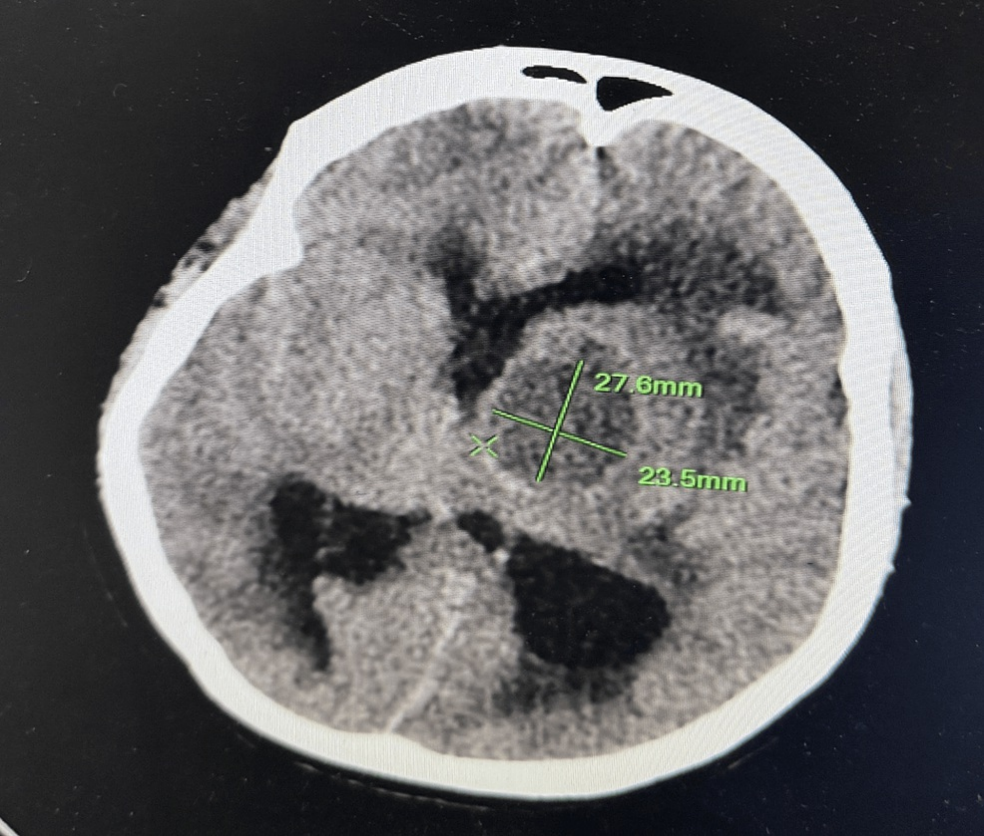
Axial NCCT scan showing AP and TV dimensions of the lesion measuring 27.6×23.5 mm, respectively. The dimensions of the abscess are marked with green lines: 27.6 mm in the AP direction and 23.5 mm in the TV direction. NCCT, non-contrast-enhanced computed tomography

**Figure 3:**
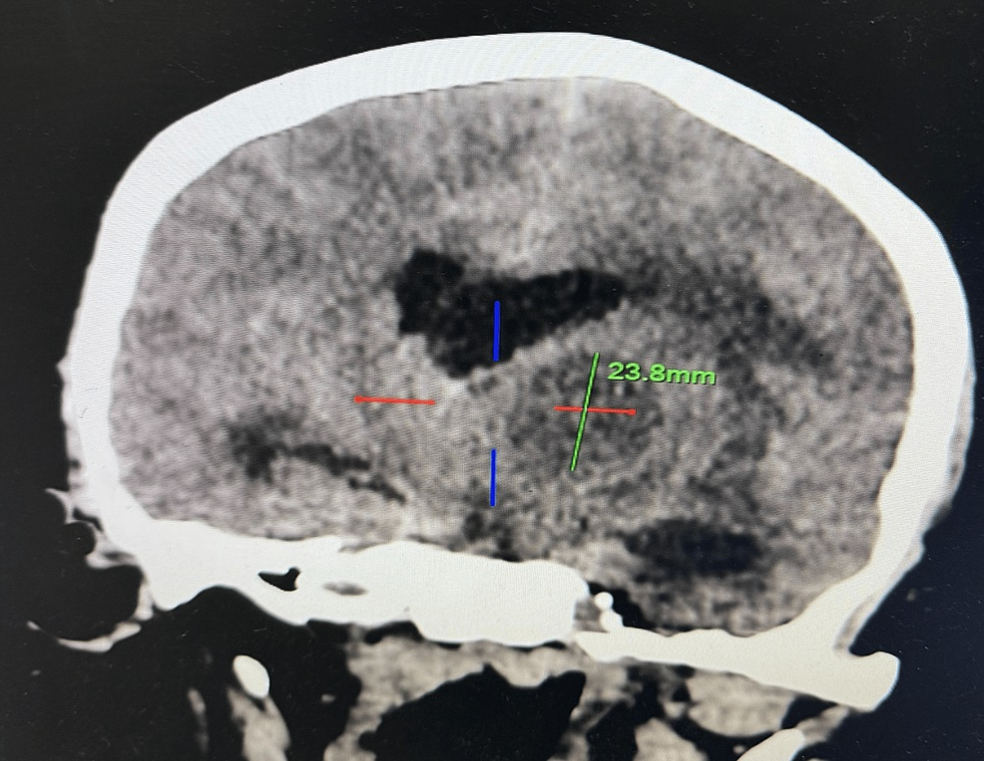
Sagittal NCCT scan showing the CC dimension of the lesion measuring 23.8 mm. The dimension of the abscess is marked with a green line: 23.8 mm in the CC direction. The red line denotes the horizontal boundaries, and the blue line represents the vertical axis through the center of the abscess. NCCT, non-contrast-enhanced computed tomography

Laboratory investigations were within normal limits except for elevated leukocyte count and C-reactive protein. No apparent cardiomegaly was found on the chest X-ray. Echocardiography evaluation revealed findings consistent with TOF, a ventricular septum defect with overriding of aorta, right ventricular hypertrophy, and thickened pulmonic valve with restricted movement suggesting pulmonary valve stenosis. There was no evidence of endocarditis on echocardiography. A non-contrast-enhanced computed tomography (NCCT) scan of the brain (Figure 1) showed a well-defined hypodense lesion measuring 23.8×23.5×27.6 mm (CCxTVxAP dimensions) (Figures 2, 3) in the left perisylvian region and thalamus with surrounding cerebral edema causing midline shift toward the right with contralateral compression of the right lateral ventricle. 

Empiric antibiotic therapy with intravenous broad-spectrum antibiotics (vancomycin and ceftriaxone) was initiated, along with antipyretics. The patient underwent urgent drainage of abscess due to midline shift and the sample was sent for culture. *Streptococcus intermedius* was observed on the culture of abscess material. Based on the antibacterial susceptibility tests, maintenance with ceftriaxone and metronidazole was planned. The department of cardiology was consulted due to TOF. The patient was maintained on antibiotic treatment for six weeks.

## Discussion

TOF is a congenital cardiac condition comprising four primary cardiac developmental abnormalities. These abnormalities with an underlying right-to-left-sided shunt manifest as cyanosis in early childhood and are thus also known as CCHD [5]. Untreated TOF is associated with several complications, including growth impairment, developmental delays, secondary polycythemia, and a more severe case of infective endocarditis. Brain abscess, although rare, is a significant complication, as highlighted in this case report. 

A brain abscess is an isolated intracerebral infection that begins as a localized area of cerebritis and grows into a collection of pus surrounded by a capsule [9]. It usually results from infectious processes such as mastoiditis, orbital cellulitis, intraoral infection, or, rarely, traumatic or surgical events. Its association with CHD is usually seen in young patients [10]. In CCHD patients, particularly those with TOF, brain abscesses occur due to hematogenous spread of infection facilitated by right-to-left shunting. This bypasses the pulmonary circulation, preventing normal filtration of blood by alveolar phagocytes, and allows direct entry of pathogens into the systemic circulation. Chronic hypoxia and metabolic acidosis due to secondary polycythemia cause hypo-perfusion of the brain, allowing the pathogens to seed under such hypo-perfused regions, further predisposing patients to infections [11]. Common pathogens causing brain abscesses in adults include streptococcus species, staphylococcus species, gram-negative bacteria, and anaerobic bacteria [10]. Brain abscesses can lead to elevated intracranial pressure and carries significant morbidity and mortality. Some of the indicators linked to a poor prognosis and high mortality rates include multiple and recurrent abscesses, delayed antibiotic administration at the time of admission, meningitis, lesions large in size or near the ventricles, and failure to perform surgical aspiration [12].

Management strategies include medical and surgical approaches. Small, deep-seated abscesses (<2 cm) and cases with coexisting meningitis are often managed medically, while large abscesses (>2 cm) are usually managed with aspiration or excision, depending on the surgeon’s expertise. The antibiotic regimen should be selected carefully based on the microorganisms isolated from blood or CSF. Antibiotics, like first-generation cephalosporins, aminoglycosides, and tetracyclines, are ineffective in treating brain abscesses as they cannot cross the blood-brain barrier [10]. When treating a surgically repaired abscess, antibiotics are recommended for four to six weeks; and in case of large multiple abscesses or an abscess treated medically, they are recommended for six to eight weeks [13]. Untreated individuals have a mortality rate between 27.5% and 71% [11]. In this case, the midline shift due to the abscess necessitated urgent drainage, leading to a successful outcome.

## Conclusions

This case report highlights the rare yet critical complication associated with unrepaired TOF, the brain abscess in a 24-year-old patient. The successful diagnosis and treatment involving both surgical and medical interventions emphasize the necessity of early diagnosis and surgical correction of TOF to prevent such life-threatening infections. Timely repair of TOF is paramount in reducing severe complications like brain abscesses, thereby improving patient outcomes and reducing mortality and morbidity associated with CHDs.
